# Ultrasound for Breast Cancer Screening in High-Risk Women: Results From a Population-Based Cancer Screening Program in China

**DOI:** 10.3389/fonc.2019.00286

**Published:** 2019-04-24

**Authors:** Yong Wang, Hongda Chen, Ni Li, Jiansong Ren, Kai Zhang, Min Dai, Jie He

**Affiliations:** ^1^Department of Ultrasound, National Cancer Center, National Clinical Research Center for Cancer, Cancer Hospital, Chinese Academy of Medical Sciences and Peking Union Medical College, Beijing, China; ^2^Office of Cancer Screening, National Cancer Center, National Clinical Research Center for Cancer, Cancer Hospital, Chinese Academy of Medical Sciences and Peking Union Medical College, Beijing, China; ^3^Department of Cancer Prevention, National Cancer Center, National Clinical Research Center for Cancer, Cancer Hospital, Chinese Academy of Medical Sciences and Peking Union Medical College, Beijing, China; ^4^Department of Thoracic Surgery, National Cancer Center, National Clinical Research Center for Cancer, Cancer Hospital, Chinese Academy of Medical Sciences and Peking Union Medical College, Beijing, China

**Keywords:** ultrasound, early diagnosis, breast cancer, cancer screening, risk prediction

## Abstract

**Background:** Ultrasound is an important modality for breast cancer screening. However, the evidence on the effectiveness of ultrasound screening in population-based cancer screening program in lacking. We aimed to evaluate the diagnostic yield of ultrasound screening in a population-based breast cancer screening in China.

**Methods:** The analyses were conducted in the context of the Cancer Screening Program in Urban China, which recruited 1,938,996 eligible participants aged 40–69 years from 16 provinces in China from 2012 to 2016. We included 72,250 women assessed to be high-risk for breast cancer who undertook ultrasound screening per study protocol. Diagnostic yield according to the Breast Imaging Reporting and Data System (BI-RADS) was evaluated. Risk factors associated with the positive findings of ultrasound were also explored by univariate and multivariable logistic regression analyses.

**Results:** Overall, there were 9,765 (13.51%) women had positive findings of ultrasound screening, including 8,487 (11.75%), 1,210 (1.67%), and 68 (0.09%) of BI-RADS categories of III, IV, and V, respectively. Younger ages, late age of 1st live birth and short-term breast feeding were found to be positively associated with positive findings under ultrasound in multivariate analyses stratified by menopause status and family history of breast cancer. Multivariable prediction models were constructed and yielded only modest prediction accuracy, with AUCs around 0.55.

**Conclusions:** We found the diagnostic yield of ultrasound screening for breast cancer in high-risk population was satisfactory. Prediction models based on environmental risk factors had limited prediction accuracy and need to be improved in the future.

## Introduction

With an estimate of 2,088,849 newly diagnosed cases in 2018 worldwide, breast cancer is the most frequently diagnosed cancer for women and is also the leading cause of cancer-related deaths (1). In China, the burden of breast cancer increased dramatically for the past decades, with incidence and mortality of 28.77 per 10,000 and 6.35 per 10,000, respectively in 2014 (2). While advantages in treatment have improved the overall outcomes of breast cancer, evidences from observational studies and randomized controlled trials have clearly demonstrated the effectiveness of breast cancer screening in reducing the mortality of breast cancer (3–5).

In most cancer screening programs, mammography was regarded as main screening method. However, the diagnostic accuracy of mammography for breast cancer was not equal in all women. The overall sensitivity of mammography for detecting breast cancer was around 85%, but it dropped dramatically to 47.8–64.4% for women with dense breast tissue (6). Previous studies have demonstrated that women with dense breast had an elevated risk of breast cancer (7). Therefore, such limitation of mammography may limit the its screening efficacy in population having a high proportion of dense breast. Ultrasound has the potential of detecting small nodules and is also widely accessible and affordable in countries having limited and unbalanced health resources (8–10). The current breast cancer screening guidelines recommended that ultrasound could be served as an auxiliary screening method to mammography (5, 11). However, most previous studies were conducted in western populations, evidences regarding the suitable screening methods in Chinese population are sparse.

Since October 2012, the China government initiated a population-based Cancer Screening Program in Urban China (CanSPUC), in which breast cancer screening is a major component. For the present study, we reported the results of breast cancer screening using ultrasound conducted between October 2012 and October 2016. We aimed to evaluate the diagnostic yield of ultrasound screening in high-risk Chinese populations and to identify risk factors associated with the clinical findings of ultrasound screening.

## Methods

### Study Design and Study Population

We performed a cross-sectional study under the framework of Cancer Screening Program in Urban China (CanSPUC). CanSPUC is an ongoing national cancer screening program which was initiated in October 2012. Briefly, residents aged 40–69 years old living in the selected communities of the participating cities were approached by trained staffs by means of phone-calls and personal encounter. After obtaining signed written informed consent, all the eligible participants were interviewed by trained staffs to collect information about their exposure to risk factors and to evaluate their cancer risk using an established risk score system. For the present screening program, to optimize use of the limited healthcare resources and to enhance the detection rate of positive findings, only participants who were assessed to be at high-risk of breast cancer were recommended to undergo subsequent ultrasound and/or mammography intervention at tertiary-level hospital designated by the program at free of charge. The study was approved by the Ethics Committee of National Cancer Center/Cancer Hospital, Chinese Academy of Medical Sciences and Peking Union Medical College and all participants provided written informed consent.

The overall screening strategy for breast cancer in this program was tailored according the age. For participant aged 40–44 years old, ultrasound was provided firstly and only those with suspicious findings under ultrasound (BI-RADS categories of III, IV, and V) were recommended to take subsequent mammography examination. For participants aged 45–69 years old, both ultrasound and mammography were provided to the participants. For patients with positive findings, further treatment were suggested according the up-to-date clinical guidelines.

**Figure 1:**
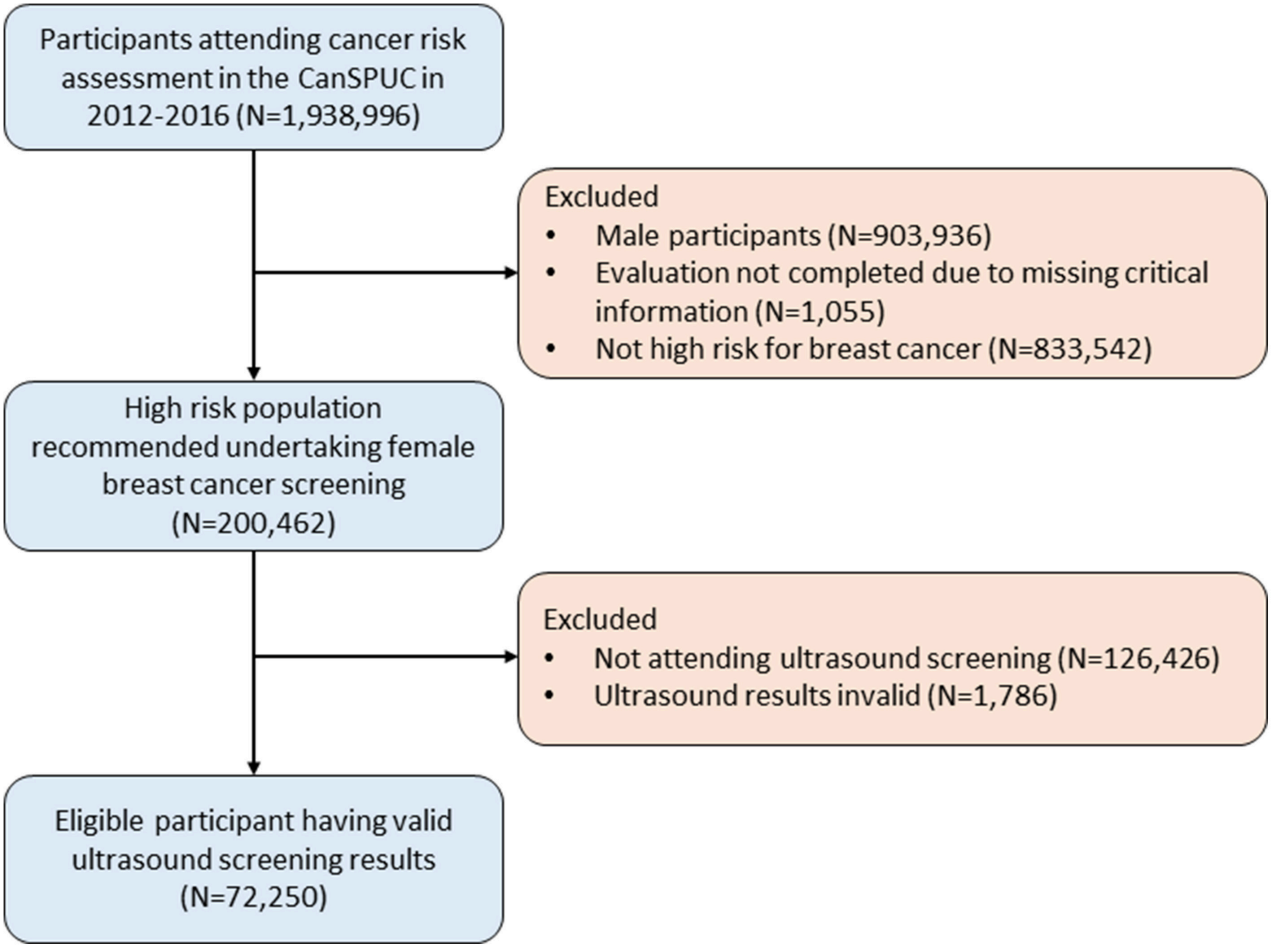
Flow diagram of the study participants in the CanSPUC from 2012 to 2016.

For the present analyses, we only used the data of the ultrasound screening conducted in the first 4 years between October 2012 and October 2016, which covered a total of 22 cities in 16 provinces. Overall, there were 1,938,996 eligible participants recruited. After excluding participants of male sex (*N* = 903,936), participants with invalid risk assessment results (*N* = 1,055), and those not at high-risk for breast cancer (*N* = 833,542), 200,462 participants were evaluated to be high-risk for breast cancer. We further excluded 126,426 participants who did not attend ultrasound screening and 1,786 participants with invalid ultrasound results, yielding an overall of 72,250 participants included in the final analyses. A flow-diagram showing the recruitment of study population is shown in the Figure 1.

### Risk Assessment

Participants were required to undertake risk assessment before clinical intervention. The rationale of the development of the cancer risk score system basically followed the Harvard Risk Index, but the included risk factors, relative risks and exposure rates of risk factors were adjusted according to the characteristics of Chinese population. Briefly, the following factors were included in the risk score system, including Body Mass Index (BMI), age of menarche, total years of menstruation, age of first marriage, total months of breastfeeding, history of breast benign diseases, History of female reproductive system surgery, and family history of breast cancer. Each risk factor was allocated a score by the expert panel based on the magnitude of its association with breast cancer. The cumulative risk scores were calculated and were then divided by the average risk score in general population to get the final individual relative risks. Individuals with relative risks over 1.50 were defined as high-risk for breast cancer.

### Clinical Procedures

Screening ultrasound was performed by color Doppler and high-resolution transducers scanning for transverse and sagittal planes by experienced radiologists (attending physician or above having experiences of endoscopy for at least 5 years). Any findings during ultrasound examination were required to be photo documented. Clinical information such as morphology, thickness, and structure of gland, features of breast space occupying solid lesions (position, size, margin, echogenicity, etc.) and clinical diagnosis were collected and documented in the data system. In this study, the Breast Imaging Reporting and Data System (BI-RADS) was used to interpret the ultrasound screening results and derive diagnosis reports, with the following categories: I, negative; II, benign; III, probably benign; IV, suspicious malignancy; and V, highly suggestive of malignancy.

For quality and consistency among all study sites, central capacity training programs were conducted annually to ensure that uniform standard of BI-RADS was implemented by radiologists from all participating hospitals. In addition, the images of all positive findings (BI-RADS categories of III, IV, and V) and 1% of randomly selected negative findings (BI-RADS categories of I and II) were centrally reviewed by an expert panel from National Cancer Center. Any discrepancy with the original diagnosis were discussed until consensus were reached.

### Data Acquisition

Paper-based standardized documentation forms (epidemiological questionnaire, clinical ultrasound examination forms) were filled by trained study staffs and physicians. Validity of forms were checked and entered into the data management system by trained study staffs. Consistency check was conducted, and mistakes were corrected by retrieving the original records if inconsistencies were identified. Each participant had a unique identification code using to track all the individual's relevant documentation forms. All data were transmitted to the Central Data Management Team, who were responsible data monitoring and subsequent data analyses.

### Statistical Analysis

Descriptive statistically analyses regarding the characteristics of the study population were firstly performed. The distribution of risk factors by different BI-RADS categories (I/II, III, and IV/V) were presented. Chi-square tests were used to compare the distribution of risk factors between participants with or without positive findings (BI-RADS category of III-V) under ultrasound screening. Multivariable logistic regression models stratified by the menopause status (pre-menopause or post-menopause) were employed to explore the associations between the risk factors and positive findings of screening ultrasound, and odds ratios (ORs) and their 95% confidence intervals (95% CIs) were also calculated and reported. Receiver operating characteristics (ROC) curves were constructed to estimate the diagnostic accuracy of multivariate logistic model using the selected risk factors for predicting the abnormal findings (BI-RADS II-IV) under screening ultrasound. Area under the curves (AUCs) along with the 95% CIs were also calculated and reported. All statistical analyses were performed with the statistical software R version 3.5.1. All tests were two-sided and *p*-values of 0.05 or less were considered to be statistically significant.

## Results

### Characteristics of the Study Population

Overall, 72,575 participants having valid ultrasound screening results were included in our analyses. Table 1 shows the sociodemographic characteristics of the study population. The mean age of the participants was 52.8 years old, with the proportions of 40–49, 50–59, and 60–69 years of 37.4, 41.4, and 21.2%, respectively. 94.9% of the population were ethnic Han, and most of the participants had an education background equal or higher than middle school, and nearly all the participants had a history of marriage.

**Table 1:** Study population characteristics among participants having breast ultrasound screening in the CanSPUC in 2012–2016.

**Group**	***N***	**Percentage (%)**
**AGE (YEARS)**		
40–49	27,018	37.4
50–59	29,946	41.4
60–69	15,286	21.2
**RACE**		
Han	68,600	94.9
Minorities	3,650	5.1
**EDUCATION**		
Primary school or below	8,271	11.5
Middle/high school	47,099	65.5
College or above	16,519	23.0
**MARITAL STATUS**		
Single	777	1.1
Married/have married	71,067	98.9
**BI-RADS CATEGORY**		
I	40,458	56.00
II	22,027	30.49
III	8,487	11.75
IV	1,210	1.67
V	68	0.09

Regarding the clinical diagnosis of ultrasound screening, 56.00% of the participants (*N* = 40,458) had negative findings (BI-RADS I). For the participants with abnormal findings, the proportions of BI-RDAS categories of II (benign), III (probably benign), IV (suspicious malignancy), and V (highly suggestive of malignancy) were 30.49% (*N* = 22,027), 11.75% (*N* = 8,487), 1.67% (*N* = 1,210), and 0.09% (*N* = 68), respectively For the nodule findings, the mean sizes for patients with BI-RADS categories of II, III, IV, and V were 5.00, 6.57, 8.58, and 16.31 mm, respectively.

### Factors Associated With BI-RADS Diagnosis of Ultrasound

We further explored the association of risk factors with BI-RADS diagnosis of ultrasound screening. Results of univariate analyses are shown in Table 2. Overall, women with high BMI (≥24.0), late age of menarche (>13 years old), at stage of pre-menopause, late age of first live birth (≥ 28 years old), and short period of breast feeding (<4 months) were tending to have abnormal findings under ultrasound screening.

**Table 2:** Distribution of risk factors among participants with different BI-RADS findings under ultrasound screening in the CanSPUC in 2012–2016.

**Factors**	**BI-RADS I, II (*N*, %)**	**BI-RADS III (*N*, %)**	**BI-RADS IV, V (*N*, %)**	*p*-value^*^
**AGE**				
40–44	8,766 (14.0)	1,414 (16.7)	177 (13.8)	<0.001
45–49	13,861 (22.2)	2,461 (29.0)	339 (26.5)	
50–54	14,617 (23.4)	2,321 (27.3)	315 (24.6)	
55–59	11,305 (18.1)	1,185 (14.0)	203 (15.9)	
60–69	13,936 (22.3)	1,106 (13.0)	244 (19.1)	
**BMI**				
<24.0	32,073 (51.4)	4,757 (56.2)	685 (53.7)	<0.001
24.0–27.9	22,911 (36.7)	2,885 (34.1)	452 (35.4)	
≥28.0	7,367 (11.8)	826 (9.8)	139 (10.9)	
**AGE OF MENARCHE**				
<13	8,872 (16.4)	1,316 (17.8)	199 (17.9)	<0.001
13–16	38,549 (71.4)	5,283 (71.6)	792 (71.3)	
>16	6,569 (12.2)	776 (10.5)	120 (10.8)	
**MENOPAUSE STATUS**				
Pre-menopause	27,553 (44.1)	3,599 (42.4)	589 (46.1)	<0.001
Post-menopause	34,932 (55.9)	4,888 (57.6)	689 (53.9)	
**TOTAL YEAR OF MENSTRUATION**				
<30	9,274 (15.0)	1,315 (15.6)	179 (14.2)	<0.001
≥30	52,745 (85.0)	7,107 (84.4)	1,085 (85.8)	
**AGE OF FIRST LIVE BIRTH**				
<28	43,351 (69.4)	5,936 (69.9)	905 (70.8)	<0.001
≥28	14,368 (23.0)	1,989 (23.4)	278 (21.8)	
Nulliparous	4,766 (7.6)	562 (6.6)	95 (7.4)	
**TOTAL MONTHS OF BREASTFEEDING**				
≥4 months	47,215 (75.6)	6,380 (75.2)	934 (73.1)	<0.001
<4 months	4,012 (6.4)	469 (5.5)	85 (6.7)	
No feeding	11,258 (18.0)	1,638 (19.3)	259 (20.3)	
**FAMILY HISTORY OF BREAST CANCER WITHIN 2-DEGREE RELATIVES**				
No	28,829 (46.1)	4,924 (58.0)	633 (49.5)	<0.001
Yes	33,656 (53.9)	3,563 (42.0)	645 (50.5)	

* *Chi-square tests comparing the distribution of risk factor groups between participants with or without positive findings (BI-RADS III-V) under ultrasound screening*.

As family history of breast cancer was an important risk factor for identifying high-risk population of breast cancer, about one half of high-risk population identified reported to have a family history of breast cancer among 2-degree relatives, and menopause status was an important predeterminant factor defining the weight of risk factors. We therefore conducted multivariable logistic regression analyses stratified by these two factors to explore the association between the risk factors with positive findings under ultrasound screening and detailed results are shown in Table 3.

**Table 3:** Associations between risk factors and positive findings (BI-RADS III-V) of breast ultrasound screening.

**Factors**	**Pre-menopause**				**Post-menopause**			
	**Without family history of breast cancer within 2-degree relatives**		**With family history of breast cancer within 2-degree relatives**		**Without family history of breast cancer within 2-degree relatives**		**With family history of breast cancer within 2-degree relatives**	
	**OR (95% CI)**	***p*-value**	**OR (95% CI)**	***p*-value**	**OR (95% CI)**	***p*-value**	**OR (95% CI)**	***p*-value**
**AGE**								
60–69	*Ref*.		*Ref*.		*Ref*.		*Ref*.	
40–49	2.18 (1.52–3.28)	<0.001	3.25 (1.89–6.18)	<0.001	1.35 (1.07–1.69)	<0.001	*1.90 (1.56–2.30)*	*<0.001*
50–59	1.92 (1.32–2.90)	<0.001	3.35 (1.94–6.37)	<0.001	1.32 (1.17–1.50)	<0.001	*1.49 (1.36–1.65)*	*<0.001*
**BMI**								
<24.0	*Ref*.		*Ref*.		*Ref*.		*Ref*.	
24.0–27.9	0.85 (0.78–0.94)	0.001	0.90 (0.82–0.99)	0.037	0.89 (0.79–1.01)	0.083	0.94 (0.86–1.04)	0.237
≥28.0	0.82 (0.71–0.97)	0.018	0.62 (0.52–0.72)	<0.001	0.95 (0.80–1.12)	0.557	0.82 (0.70–0.96)	0.012
**AGE OF MENARCHE**								
>16	*Ref*.		*Ref*.		*Ref*.		*Ref*.	
13–16	1.01 (0.86–1.19)	0.900	0.97 (0.83–1.15)	0.761	1.05 (0.89–1.23)	0.572	1.11 (0.98–1.29)	0.113
<13	0.93 (0.77–1.12)	0.424	1.16 (0.96–1.41)	0.118	1.19 (0.96–1.48)	0.111	1.11 (0.93–1.32)	0.254
**TOTAL YEAR OF MENSTRUATION**								
<30	*Ref*.		*Ref*.		*Ref*.		*Ref*.	
≥30	1.01 (0.90–1.13)	0.854	1.27 (1.12–1.45)	<0.001	0.98 (0.82–1.17)	0.788	1.01 (0.85–1.20)	0.944
**AGE OF 1ST LIVE BIRTH**								
<28	*Ref*.		*Ref*.		*Ref*.		*Ref*.	
≥28	1.11 (1.01–1.24)	0.030	1.02 (0.92–1.14)	0.703	0.90 (0.79–1.04)	0.157	1.02 (0.92–1.14)	0.682
Nulliparous	1.15 (0.63–1.20)	0.633	0.89 (0.67–1.19)	0.444	0.54 (0.34–0.82)	0.005	1.39 (0.75–2.48)	0.283
**BREAST FEEDING**								
≥4 months	*Ref*.		*Ref*.		*Ref*.		*Ref*.	
<4 months	1.09 (0.61–2.01)	0.778	0.92 (0.67–1.26)	0.589	1.33 (0.84–2.13)	0.231	0.62 (0.33–1.18)	0.147
No feeding	1.12 (1.01–1.24)	0.032	1.06 (0.93–1.20)	0.371	1.08 (0.91–1.28)	0.371	1.03 (0.92–1.15)	0.626

*OR, odds ratio; 95% CI, 95% confidence interval*.

For pre-menopause women without family history of breast cancer within 2-degree relatives, age, BMI, age of 1st live birth and duration of breast feeding were found to be associated with the positive findings of breast ultrasound screening. For instance, compared to women aged of 60–69 years old, women aged of 40–49 years old, and 50–59 years old had higher likelihood to have positive findings of ultrasound, with ORs of 2.18 (1.52–3.28) and 1.92 (1.32–2.90), respectively. For pre-menopause women with family history of breast cancer, age, BMI and total years of menstruation were also found to be associated factors. Similarly, for post-menopause women without or with family history of breast cancer within 2-degree relatives, age were also identified to be a potential risk factor associated with the abnormal findings of ultrasound screening.

### Models for Predicting Abnormal Findings Under Ultrasound Screening

**Figure 2:**
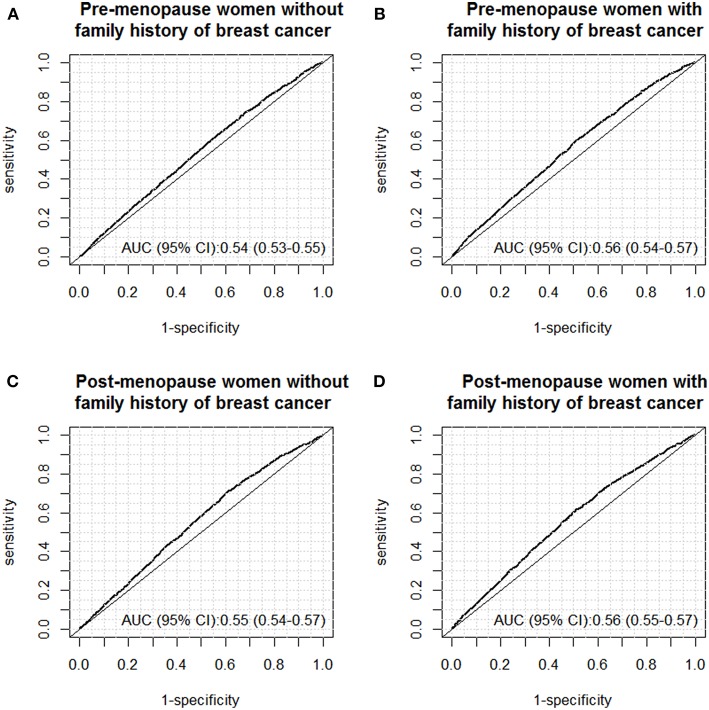
ROC curves of the models for predicting abnormal findings (BI-RADS III-V) under breast ultrasound screening in the following subgroups: **(A)** pre-menopause women without family history of breast cancer; **(B)** pre-menopause women with family history of breast cancer; **(C)** post-menopause women without family history of breast cancer; **(D)** post-menopause women with family history of breast cancer.

By using the above-mentioned risk factors, we further constructed multivariable logistic regression models to predict abnormal findings under ultrasound screening for the four subgroups, and the diagnostic accuracy was evaluated by constructing ROC curves. The results of ROC curves are shown in Figure 2. Overall, the four prediction models only yielded poor diagnostic accuracy for prediction women with abnormal findings under ultrasound examinations, with AUCs around 0.55. For instance, for premenopausal women without family history of breast cancer within 2-degree relatives, the AUC was 0.54 (95% CI: 0.53–0.55). Similar AUCs were also observed for the rest three subgroups.

## Discussion

We reported here the preliminary results of 72,575 high-risk women who undertook ultrasound screening in a population-based cancer screening program in China. The analyses showed that positivity rate for abnormal findings (BI-RADS III-V) of ultrasound screening in this high-risk population was 13.51%, with BI-RADS categories III, IV, and V of 11.75, 1.67, and 0.09%, respectively. Additionally, we identified several factors including age, BMI, age of first live birth and duration of breast feeding were potentially associated with the positive findings of ultrasound. However, multivariable prediction models using these factors only conferred modest diagnostic performance. To our limited knowledge, this is the first large-scale study reporting the diagnostic findings of ultrasound screening in a population-based cancer screening program in China. The finding of our study provided timely estimate of screening yield of ultrasound in breast cancer screening programs and will be helpful for designing effective breast cancer screening strategies in future.

Ultrasound was suggested to serve as an adjunctive screening method for women having dense breasts (3). Regarding the epidemiology of dense breasts, about 25 million women (about 43.3%) aged 40–74 years are classified as having heterogeneously or extremely dense breasts according to data from Breast Cancer Surveillance Consortium in the US (12). For Chinese women, the prevalent of dense breast was even higher which therefore further limited the efficacy of mammography in breast cancer screening (13, 14). One previous research in China also demonstrated that ultrasound was superior to mammography for breast cancer screening in high-risk Chinese women (15). Therefore, ultrasound was regarded as an important adjunctive method to mammography for breast cancer screening in Chinese population.

In our study, the detection rate for suspicious malignancy (BI-RADS IV and V) was 0.17%. Our results were in line with previous researches conducted in China (15, 16). As active and passive follow-ups collecting the health outcomes of the participants is still under way in this cancer screening program, sensitivity, specificity, and positive/negative predictive values of the ultrasound for detecting breast cancer cannot be assessed in the current analyses and will be explored in further research.

Previous studies have identified a series of risk factors of breast cancer and risk prediction models based on such risk factors such as the Gail breast cancer risk model have been developed to identified women at high risk for breast cancer for preventive interventions or more intensive surveillance (17–21). In our study, we found BMI, age of menarche, age of first live birth and duration of breast feeding were associated with the positive findings under ultrasound examinations, which were lines with previous studies. As some factors (such as age of menarche) were also included in the risk score system to select high risk population, the magnitude of association might be underestimated. However, such analyses are indispensable to validate pre-included factors and explore new risk factors, with the purpose of further optimizing the risk assessment model for future research.

It deserves to be noted that the overall positivity rate of ultrasound screening was high in a high-risk population in urban China, with around 44% participants having benign or potential malignancies. Therefore, the potential harms of ultrasound screening including the consequences of false-positive and false-negative tests, and the occurrence of over-diagnosis cannot be neglected. Further studies addressing the estimates of the positive and negative effects of ultrasound screening in women based on the latest evidence are required to help policy makers in their decision-making about implementation of the breast cancer screening programs. Although ultrasound had several advantages over mammography such as lower cost and easier accessibility especially in resource poor regions, it had barriers in screening programs, such as operator dependence procedure, limited ability to detect calcifications, lack of trained technologist, and limited reproducibility. Further large-scale trials and rigorous health-economic evaluations should be conducted to illustrate whether ultrasound screening is cost-effective in breast cancer screening programs.

Specific strengths and limitations deserve careful attention when interpreting our results. A major strength of our study is the fact that our analyses were the first to illustrate diagnostic yield of ultrasound screening in a large-scale population-based cancer screening program in China. Furthermore, detailed patient information including epidemiological questionnaire and clinical examination data were collected in a standardized manner by trained study staffs to ensure the quality of data. Capacity training and central review of ultrasound reports by an expert panel were also conducted yearly to enhance the consistency and accuracy of clinical diagnoses. Limitations include that the study population was a pre-selected high-risk population using the predefined risk model which was not representative of entire general population of China and therefore selection bias cannot be ruled out. In addition, follow-ups tracing the outcomes of all the participants are undergoing, so evaluation of detection rate of breast cancer or occurrent of interval cancer cannot be evaluate at the current stage.

In summary, in this large-scale cancer screening program in China, we found the diagnostic yield of ultrasound screening for breast cancer in high-risk population was satisfactory. Using environmental risk factors associated with positive findings of ultrasound identified in this study only carried limited prediction accuracy and further improvement by incorporating other effective factors could contribute to the development of a useful risk prediction model for identifying high-risk populations of breast cancer in the future.

## Ethics Statement

The study was approved by the Ethics Committee of National Cancer Center/Cancer Hospital, Chinese Academy of Medical Sciences and Peking Union Medical College and all participants provided written informed consent.

## Author Contributions

JH and MD: conception and design. YW and HC: statistical analyses and drafting of the article. YW, HC, NL, JR, and KZ: data acquisition and data interpretation. All authors revised the manuscript and approved the final version of the manuscript.

### Conflict of Interest Statement

The authors declare that the research was conducted in the absence of any commercial or financial relationships that could be construed as a potential conflict of interest.
